# PP64 The Problem Of Applying Risk-Of-Bias Tools In Studies Of Medical Devices: A Case Study In Hypertension

**DOI:** 10.1017/S0266462325102833

**Published:** 2025-12-29

**Authors:** Alice Sanderson, Liesl Strachan, Silke Walleser Autiero, Rachael McCool

## Abstract

**Introduction:**

Assessing the risk of bias (RoB) using validated tools is an important aspect of any health technology assessment (HTA). However, many of these tools were designed with pharmaceuticals in mind. Well conducted trials of medical devices often face different challenges. Consequently, choosing the RoB tool to use in any assessment may play a large role in how evidence of such technologies is perceived.

**Methods:**

A systematic review and meta-analysis was conducted on renal denervation devices for treating uncontrolled hypertension. Only randomized controlled trials (RCTs) were included, so the revised Cochrane RoB tool was chosen to assess bias in individual studies. RoB assessments were undertaken by two reviewers.

**Results:**

Twenty-five RCTs met the eligibility criteria for the systematic review and were assessed for RoB. Application of the revised Cochrane RoB tool resulted in some disparity in the final assessments, compared with the quality criteria for RCTs defined in previously published clinical guidelines for hypertension. Specifically, some of the sham-controlled RCTs had an overall RoB rating of “some concerns”, whereas some of the open-label RCTs had a low RoB and the tool seemed unable to distinguish between the designs. Given that sham-controlled trials can control for a potential placebo effect, this result was both unexpected and unintuitive.

**Conclusions:**

Designing RCTs for medical devices requires substantial input, planning, and consideration from multiple stakeholders. Assessing the RoB of such studies, although necessary from a decision-maker perspective, requires careful contextual interpretation to understand and fully appreciate the nuances at play. Potential for a new RoB tool for assessing studies of medical devices is warranted.

